# *PTBP3* modulates P53 expression and promotes colorectal cancer cell proliferation by maintaining *UBE4A* mRNA stability

**DOI:** 10.1038/s41419-022-04564-8

**Published:** 2022-02-08

**Authors:** Canbin Xie, Fei Long, Liang Li, Xiaorong Li, Min Ma, Zhixing Lu, Runliu Wu, Yi Zhang, Lihua Huang, Jing Chou, Ni Gong, Gui Hu, Changwei Lin

**Affiliations:** 1 Department of Gastrointestinal Surgery, The Third XiangYa Hospital of Central South University, Changsha, Hunan 410013 China; 2 Center for Experimental Medicine, The Third XiangYa Hospital of Central South University, Changsha, Hunan 410013 China; 3 School of Life Sciences, Central South University, Changsha, Hunan 410078 China

**Keywords:** Cancer genomics, Oncogenes, Tumour biomarkers

## Abstract

The RNA binding protein *PTBP3* was recently reported to play a critical role in multiple cancers, and the molecular mechanisms involved RNA splicing, 3′ end processing and translation. However, the role of *PTBP3* in colorectal cancer (CRC) remains poorly explored. Herein, *PTBP3* was upregulated in CRC and associated with a poor prognosis. *PTBP3* knockdown in colorectal cancer cell lines restricted CRC proliferative capacities in vitro and in vivo. Mechanistically, *PTBP3* regulated the expression of the E3 ubiquitin ligase *UBE4A* by binding the 3′ UTR of its mRNA, preventing its degradation. *UBE4A* participated in P53 degradation, and *PTBP3* knockdown in colorectal cancer cell lines showed increased P53 expression. *UBE4A* overexpression rescued *PTBP3* knockdown-induced inhibition of CRC cell proliferation and P53 expression. Our results demonstrated that *PTBP3* plays an essential role in CRC cell proliferation by stabilizing *UBE4A* to regulate P53 expression and may serve as a new prognostic biomarker and effective therapeutic target for CRC.

## Introduction

Colorectal cancer (CRC) is the most common malignancy worldwide and the second leading cause of cancer-related death worldwide [1, 2]. Although survival has improved because of advances in surgical techniques, malignant growth and metastasis remain major causes of cancer-related death [3]. Therefore, increasing our understanding of the mechanisms that drive CRC progression is urgent.

*PTBP3* was first identified as an essential RNA-binding protein in 1999 [4] and is a member of the PTB family containing three paralogs—namely, *PTBP1*, *PTBP2* and *PTBP3* [5]. Previous studies have shown that *PTBP3* is dysregulated and promotes the progression of numerous cancers, including breast cancer [6], hepatocellular cancer [7], gastric cancer [8] and CRC [9]. The functions of *PTBP3* include RNA splicing, translational activation and mRNA stability [6–10]. However, the specific function of *PTBP3* and its potential mechanism in CRC proliferation remain largely unknown.

E3 ubiquitin ligase ubiquitination factor E4A (*UBE4A*) is a U-box-containing ubiquitylation enzyme [11]. Similar to *UBE4B*, *UBE4A* belongs to the human homologs of the yeast *UFD2* ubiquitination factor family, whose members share a conserved domain of approximately 70 amino acids named the U box [12–14]. Previous studies have implicated *UBE4B* as a regulator of P53 [15–18]; the U box catalytic domain is closely related to the RING-finger domain of *MDM2* and is responsible for its E3 activity. These two enzymes are considered significant regulators of P53 through the ubiquitination process [17]. However, the function of *UBE4A*, as a U box catalytic domain-containing E3 ubiquitin ligase, remains largely unknown. Previous studies have reported that PCBP1, BMP7 and Viperin are *UBE4A*-ubiquitinated substrates [19–21]. In gastrointestinal disease, *UBE4A* dysregulation in enteroendocrine cells is associated with Crohn’s disease [22], but its function and regulatory mechanism in CRC remain unclear.

In this study, we attempted to identify the function and regulatory mechanism of *PTBP3* in CRC. *PTBP3* was upregulated in CRC patients with a poor prognosis and led to CRC proliferation, suggesting that *PTBP3* may be a crucial factor in CRC development. Mechanistically, *PTBP3* regulated the expression of the E3 ubiquitin ligase *UBE4A* by binding to its 3′ untranslated region (UTR) to prevent its mRNA degradation. Additionally, *UBE4A* promoted CRC progression and participated in P53 degradation. Our results provide new evidence that *PTBP3* exerts an oncogenic function and that the *PTBP3/UBE4A/*P53 axis might serve as a potential therapeutic target for CRC.

## Materials and methods

### Tissue collection and ethics statement

Clinical material was obtained from patients treated at the Third Xiangya Hospital of Central South University (Changsha, China) with informed consent and approval of the Medical Ethics Central South University(No:2020-S095). Tissue specimens were snap frozen and stored in liquid nitrogen until further use.

### Cell lines and cell culture

All colorectal cancer cell lines were purchased from KeyGEN BioTECH (Jiangsu, China). FHC was purchased from American Type Culture Collection (ATCC) (Manassas, Virginia, USA). All the cell lines were authenticated using STR (or SNP) profiling within the last 3 years. All the experiments were performed using mycoplasma-free cells. Colorectal cancer SW620 (RRID: CVCL_0547) and SW480 (RRID: CVCL_0546) cells were cultured in L15 (KeyGEN BioTECH, Jiangsu, China) medium supplemented with 10% fetal bovine serum (FBS; Biological Industries, Israel) and 1% antibiotics (100 U/ml of penicillin and 100 mg/ml of streptomycin; Life Technologies, Inc., Grand Island, NY, USA). HCT116 (RRID: CVCL_0291) and HT29 (RRID: CVCL_0320) cells were cultured in McCoy’s 5 A (KeyGEN BioTECH) medium supplemented with 10% FBS and 1% antibiotics. LoVo (RRID:CVCL_0399) cells were cultured in Dulbecco’s modified Eagle’s medium (DMEM; KeyGEN BioTECH) supplemented with 10% FBS and 1% antibiotics. FHC (RRID:CVCL_3688) cells were cultured in Roswell Park Memorial Institute 1640 (RPMI 1640; KeyGEN BioTECH) medium supplemented with 10% fetal bovine serum and 1% antibiotics. All the cell lines were grown in a 5% CO_2_ cell culture incubator at 37 °C.

### Patients and tissue sampling

All the clinical material was obtained from patients who had undergone surgical resection for CRC at the Third XiangYa Hospital of Central South University (Changsha, China) after informed consent and approval from the Medical Ethics Central South University (No:2020-S095).

### Quantitative real-time PCR assays

Total RNA from cells and tissues was extracted using TRIzol Reagent (Invitrogen, Carlsbad, CA, USA). cDNA was generated using the ReverTra Ace qPCR RT Master Mix (TOYOBO, Osaka, Japan). Quantitative real-time PCR (qRT-PCR) was performed using a LightCycler 480 Real Time PCR instrument (Roche, Basel, Switzerland). GAPDH was used to normalize the qRT-PCR data. All the primer pairs were purchased from Sangon Biotech (Shanghai, China), and sequences were available in Supplementary Table 1.

### Western blot assays

Whole cells and tissues were collected and lysed using 1× RIPA buffer (KeyGEN BioTECH) containing 1% PMSF (KeyGEN BioTECH) to harvest proteins. The protein samples were separated by 10% sodium dodecyl sulfate-polyacrylamide gel electrophoresis (SDS-PAGE), transferred to polyvinylidene fluoride membranes (PVDF) (Millipore, CA, USA), blocked with 5% skim milk for 2 h, incubated with primary antibodies at 4 °C overnight and secondary antibodies for 1 h, and visualized using an Odyssey CLx Infrared Imaging System (LI-COR Biosciences, NE, USA). The antibodies used for western blotting (WB) are provided in Supplementary Table 2.

### Lentiviral vector and transfection

Lentiviruses against *PTBP3* and *UBE4A* and their corresponding negative controls were obtained from Shanghai GenePharma Co., Ltd. To generate stable lentivirus-transduced lines, cells were infected with virus and polybrene following the manufacturer’s recommendations, and stable cell lines were selected using 4 µg/ml of puromycin after 72 h of transfection. The efficiency in different cells was determined by the GFP intensity, qRT-PCR and WB. The shRNA sequences are listed in Supplementary Table 1.

### Plasmid transfection

*UBE4A* plasmids were obtained from Shanghai GenePharma Co., Ltd. After transfection with the *UBE4A* plasmid, the cells were seeded into six-well plates and cultured for 24 h. When the density reached 50–60%, Lipofectamine 3000 reagent (Invitrogen, USA) was used to transfect the *UBE4A* plasmids according to the instructions.

### Cell proliferation and colony formation assays

The CCK-8 assay (Dojindo, Kumamoto, Japan) was used to measure cell proliferation in 96-well plates. Approximately 2000 HCT116 cells, 2000 SW480 cells and 1000 LOVO cells were seeded per well, with six replicates for each condition. CCK8 was added at 0, 24, 48, and 72 h, followed by incubation at 37°C for 2 h. The absorbance values (A450) were detected using an EnVision microplate reader (PerkinElmer). For colony formation assays, approximately 100 HCT116 cells, 1000 LOVO cells and 1000 SW480 cells were seeded in each well of a 6-well plate in triplicate for each condition and incubated for 7 to 12 days. The colonies were fixed with methanol, stained with crystal violet, and counted. The average colony counts were calculated, and paired t-test was used to test statistical significance. Each experiment was repeated three times.

### Flow cytometric analysis

Cell cycle analysis was measured by flow cytometry. A total of 100000 cells labeled with propidium iodide (PI; Sigma-Aldrich, USA) were prepared from each group and analyzed using a FACSCalibur flow cytometer (BD Biosciences). Next, the proportions of G0/G1, S and G2/M cells were calculated and compared using ModFit LT 3.1 software. The results were analyzed using a FACSCalibur flow cytometer (BD Biosciences).

### Tumor xenografts

The nude mouse xenograft tumor growth model was developed according to the guidelines for experimental animal management established by Kagawa University and guidelines for the welfare and use of animals in cancer research [23]. Female BALB/c nude mice (4–5 weeks, 18–20 g) were obtained from the Department of Laboratory Animals of Central South University and maintained under specific pathogen-free conditions. We first weighed and numbered the nude mice, which were numbered 1–12 respectively. Then we wrote the numbers 1–12 on the paper, mixed them and extracted them in four groups, with three numbers in each group. The first group was HCT116 sh-NC,the second group was HCT116 sh-*PTBP3*,the third group was LoVo sh-NC,the last group was LoVo sh-*PTBP3*.Next, 3 × 10^6^ HCT116-shNC (small hairpin carrying negative control RNA) cells and HCT116-sh*PTBP3* (small hairpin carrying *PTBP3*-specific RNA) cells and 2 × 10^6^ LoVo-shNC cells and LoVo-sh*PTBP3* cells were harvested and injected subcutaneously into the left or right flank of the nude mice (*n* = 3 per group). Tumors were recorded using calipers and an electronic scale to estimate the tumor volume and weight every four days. Thirty days after the injection, the mice were killed by an overdose of pentobarbital (250 mg/kg; intraperitoneal injection), and the final tumor volume and weight results were recorded. The tumor volumes were calculated based on the formula: volume (mm3) = length (mm) × width (mm) × width (mm)/2. Tumors were further embedded in paraffin for H&E and immunohistochemistry (IHC).

### Immunohistochemistry

Tissue sections from the nude mice were embedded in paraffin and then were deparaffined and rehydrated. Next, endogenous peroxidase activity was blocked by incubating the tissue sections with 0.3% hydrogen peroxide for 20 min. After that, the tissue sections were blocked in 10% BSA for 10 min and incubated with anti-human Ki-67 (1:100) antibodies and anti-human *PTBP3* antibody (1:100) at 4 °C for 12 h. The tumor sections were then incubated in biotinylated secondary antibodies for 20 min at room temperature. After that, the tissue sections were reacted with streptavidin-peroxidase conjugate for 10 min. Next, 3,3’-diaminobenzidine was added as the chromogen substrate. Images were captured using an inverted microscope system (IX73; Olympus, Japan).

### RIP assay

RNA-binding protein immunoprecipitation (RIP) was performed using the EZ-Magna RIP Kit (Merck, KGaA, Darmstadt, Germany; Catalog No. 17–701) according to the manufacturer’s instructions. Approximately 2 × 10^7^ HCT116 and LOVO cells were washed with ice-cold PBS and resuspended in RIP lysis buffer containing a protease inhibitor mixture and RNase inhibitor. Next, magnetic bead protein A/G was incubated with 5 µg of IgG (negative control) (Merck KGaA) or *PTBP3* (Santa Cruz) antibody for 30 min at room temperature. Cell lysis buffer and immunoprecipitation buffer containing EDTA and RNase inhibitor were added to the complexes. Thereafter, the complexes were incubated with rotation overnight at 4 °C. The next day, the complex was washed with washing buffer containing proteinase K and 10% SDS and then heated at 55 °C for 30 min. Finally, RNA was extracted and purified for RT-qPCR analysis. RIP assays were performed in biological triplicates and were detected by RT-qPCR. The primers are described in Supplementary Table 1.

### RNA antisense purification assay

The RNA antisense purification assay was performed using the RNA Antisense Purification (RAP) Kit (Bersin BioTM, Guangzhou, China; CataLog Bes5103-3) according to the manufacturer’s instructions. Approximately 2 × 10^7^ HCT116 cells were washed with ice-cold PBS and resuspended in methanol and 1.375 M Glycine to cross-link cells. Next, lysis buffer containing the protease inhibitor mixture and RNase inhibitor was added for homogenization. Furthermore, DNase salt stock, DNase, EDTA, EGTA, and DTT were added to remove DNA. Thereafter, the probes were added to the processed sample, followed by hybridization at 37 °C for 30 min (the probes are described in Supplementary Table 1), incubate degeneration at 50 °C for 50 min, and hybridization again at 37 °C for 180 min. Next, streptavidin beads were added to the complex and incubated for 30 min. After washing with wash buffer, the RAP mix bound to the beads was eluted and then resuspended in 60 μLof 1× loading buffer and boiled for 5 min, followed by Western blot detection.

### Luciferase reporter assays

HCT116sh-NC cells, HCT116sh-*PTBP3* cells, Lovosh-nc cells and Lovosh-*PTBP3* cells were seeded at a density of 1 × 10^5^ cells per well in a 24-well plate. The cells were transfected with the pRL-TK (Promega) Renilla plasmid using Lipofectamine 2000 according to the manufacturer’s instructions (Invitrogen). The Renilla luciferase sequence in the pRL-TK vector (Promega, WI, USA) was used as an internal control. Dual luciferase reporter assays were performed according to the protocol using the Dual-Luciferase Reporter Assay System (cat. E1910; Promega). The firefly luciferase activity was normalized to the Renilla luciferase activity. The data were expressed as the percent of luciferase activity in control cells (100%).

### Immunoprecipitation assay

Immunoprecipitation was performed according to the manufacturer’s instructions (Thermo, PierceTM Classic Magnetic, Rockford, USA; LOT: UC283101). HCT116 cell lysates were prepared in immunoprecipitation lysis buffer (20 mM Tris-Cl, pH 8.0, 10 mM NaCl, 1 mM EDTA, 0.5% NP-40) containing a protease inhibitor cocktail (Sigma). Cell extracts (2 mg) were precleared with 50 μl of protein A/G-agarose (Santa Cruz) at 4 °C for 2 h, and the supernatant was incubated with the corresponding antibodies with gentle shaking at 4 °C overnight, followed by the addition of 50 μl of protein A/G-agarose for another 2 h. The beads were washed and then resuspended in 60 μl of 1× loading buffer, boiled for 5 min, and subjected to Western blot detection.

### Bioinformatics analysis

The *PTBP3* expression data were downloaded from TCGA (https://gdc.cancer.gov/) and GEO (https://www.ncbi.nlm.nih.gov/geo). Survival analysis of *PTBP3* was performed using the Gene Expression Profiling Interaction Analysis database (http://gepia.cancer-pku.cn/) [24]. The correlated genes of *PTBP3* were analyzed using TCGA data and starBase v2.0 (http://starbase.sysu.edu.cn/index.php) [25]. The correlated pathway of *PTBP3* was analyzed using Gene Set Enrichment Analysis [26].

### Statistical analysis

Statistical computations were performed using GraphPad Prism 8.0. (San Diego, CA). The data were presented as means ± s.d. of three independent experiments except where otherwise indicated. To compare the differences between two groups, Student’s *t*-test was performed. Two-way analysis of variance (ANOVA) was used for comparisons between the different groups. The relationship between gene expression and clinicopathological indicators was examined using chi-squared test, and *p* < 0.05 was considered statistically significant.

## Results

### *PTBP3* is upregulated in CRC, and high *PTBP3* expression correlates with a poor prognosis

**Fig. 1:**
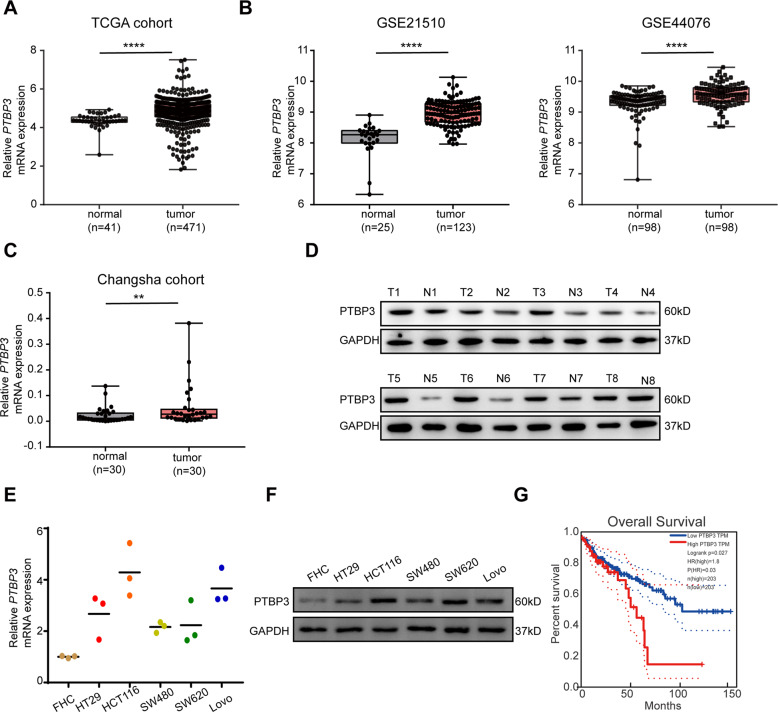
*PTBP3* is overexpressed in CRC and is associated with a poor prognosis. **A** *PTBP3* mRNA level in the TCGA database (two-tailed Student’s *t*-test, *P* < 0.0001). **B** *PTBP3* mRNA level in the GEO database (two-tailed Student’s *t*-test, *P* < 0.0001). **C** *PTBP3* mRNA levels in CRC tissues compared with those in adjacent normal tissues (*n* = 30; measured by qRT-PCR; GAPDH was used as an internal control) (two-tailed Student’s *t*-test, *P* < 0.01). **D** *PTBP3* protein levels in CRC tissues compared with those in adjacent normal tissues (*n* = 8; measured by Western blotting). **E** *PTBP3* mRNA levels in colorectal cancer cell lines (HT29, SW480, HCT116, SW620 and LoVo) compared with those in FHC cells, a normal colon cell line (measured by qRT-PCR; GAPDH was used as an internal control) (two-tailed Student’s *t*-test, *P* < 0.05). **F** *PTBP3* protein levels in colorectal cancer cell lines (HT29, SW480, HCT116, SW620 and LoVo) compared with those in FHC cells (measured by Western blotting). **G** Kaplan–Meier estimated overall survival in patients with high or low *PTBP3* expression. Group cutoff-points: 25% (high) and 75% (low). The results are presented as means ± s.d. and are representative of at least three independent experiments.

To identify the role of *PTBP3* in CRC development, we first assessed the expression of *PTBP3* in CRC tissues. The gene expression data for 471 colorectal cancer samples were downloaded from The Cancer Genome Atlas (TCGA) database. *PTBP3* expression was first analyzed in 471 CRC tissues and 41 normal colorectal tissues, and *PTBP3* was significantly upregulated in CRC tissues (Fig. 1A). Next, we downloaded and assessed two CRC gene expression datasets, GSE21510 and GSE44076, from the Gene Expression Omnibus (GEO) database, and the same trend was observed (Fig. 1B). To further confirm this conclusion, we detected *PTBP3* expression in 30 matched pairs of human CRC tissues and adjacent nontumor tissues by qRT-PCR and 8 matched pairs of tissues by western blotting (WB). *PTBP3* was significantly upregulated in human CRC tissues (Fig. 1C, D). Next, we summarized the clinicopathological characteristics of the 30 patients in the first cohort, and patients with high *PTBP3* expression showed larger tumor sizes than those with low *PTBP3* expression (Table 1). Additionally, *PTBP3* expression was detected in 5 human CRC cell lines (HT-29, HCT116, SW480, SW620 and LoVo) and normal human colonic epithelial FHC cells, and *PTBP3* expression was significantly higher in cancer cell lines than in FHC cells (Fig. 1E, F). HCT116 and LoVo cells with relatively high *PTBP3* expression were selected for subsequent functional assays. Additionally, we examined the correlation between the *PTBP3* expression level and prognosis of CRC patients using the Gene Expression Profiling Interactive Analysis (GEPIA) database. Kaplan–Meier survival analysis showed that patients with high *PTBP3* levels had shorter overall survival times than those with low *PTBP3* levels (Fig. 1G). Collectively, these results showed that *PTBP3* was overexpressed in CRC patients with a poor prognosis.

**Table 1:** Clinic-pathological characteristics of enrolled patients.

Clinicopathological characteristics	Total (*n* = 30)	*PTBP3*		
		High^a^ (*n* = 15)	low^a^ (*n* = 15)	*P* value
Mean age	63.5 ± 1.853	66.07 ± 2.792	60.93 ± 2.343	0.17
Sex				0.6903
Male	21	11	10	
Female	9	4	5	
Tumor site				0.3940
Left colon	1	0	1	
Right colon	12	5	7	
Rectum	17	10	7	
Staging				0.2557
I–II	19	11	8	
III–IV	11	4	7	
Tumor size				0.0464*
≥5 cm	11	7	2	
<5 cm	19	8	13	
Differentiation		0.9103		
CRC,WD	9	5	4	
CRC,MD	13	6	7	
CRC,PD	8	4	4	
Tumor stage				>0.9999
T1–T2	8	4	4	
T3–T4	22	11	11	
Lymph node				0.0803
N0	19	11	8	
N1	7	1	6	
N2	4	3	1	
Distant metastasis				0.3091
M0	29	15	14	
M1	1	0	1	

*Statistical significance was determined by Chi-squared test, P < 0.05.

^a^Low and high expression groups were determined by the cutoff-point 50% (15 of 30) and 50% (15 of 30) of *PTBP3* in 30 tumor tissue specimens.

### *PTBP3* is required for the proliferation of CRC cells in vitro and in vivo

**Fig. 2:**
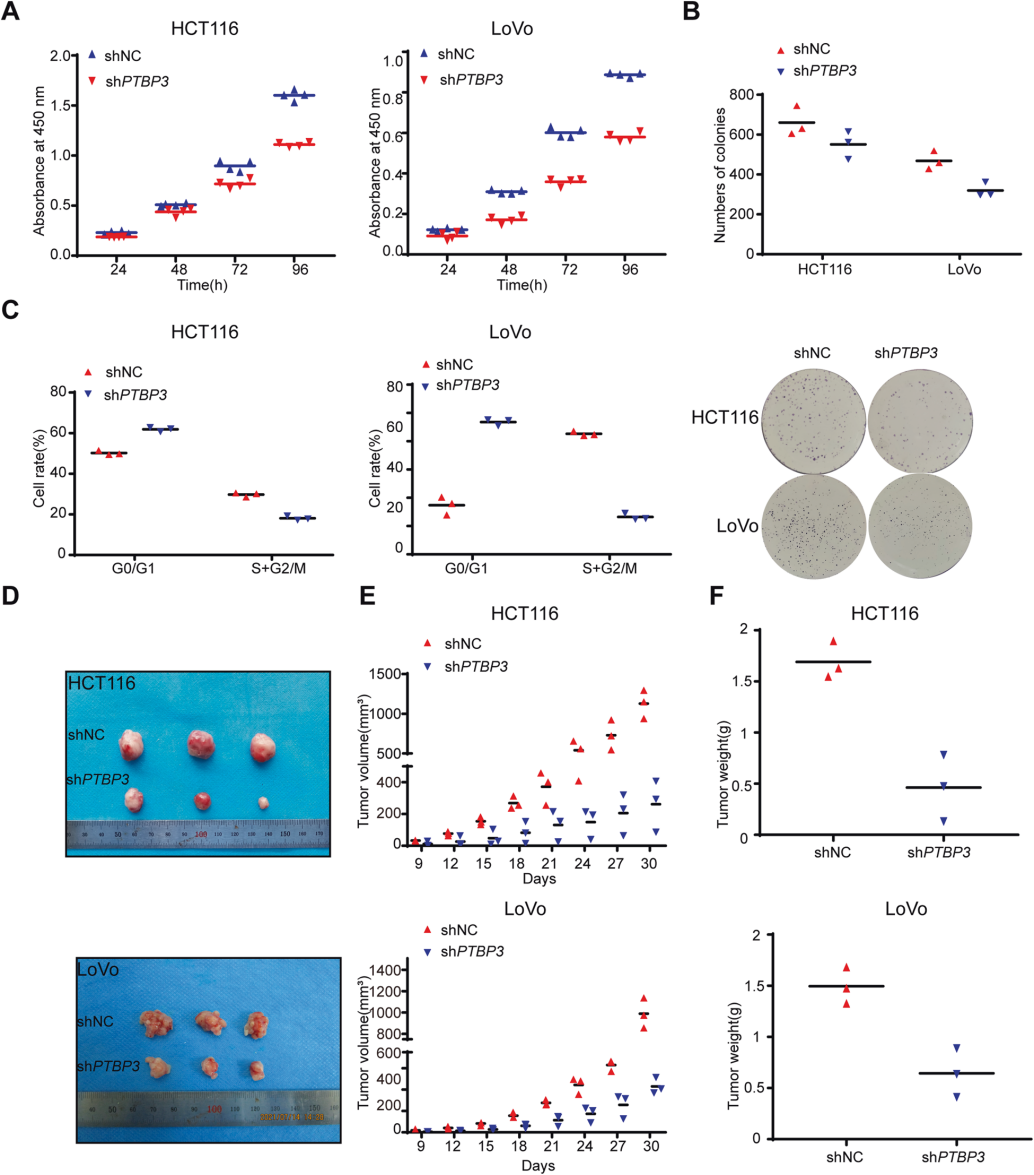
*PTBP3* promotes CRC cell proliferation in vitro and in vivo. **A** Reduction in the proliferation ability of sh*PTBP3* HCT116 and LoVo cells compared with that of control (NC) cells using the CCK-8 assay (two-way ANOVA, *P* < 0.01). **B** Reduction in the colony formation ability of sh*PTBP3* HCT116 and LoVo cells compared with that of control (NC) cells using the colony formation assay. The bar graph indicates the number of colonies (two-tailed Student’s *t*-test, *P* < 0.01). **C** Cell cycle distribution of sh*PTBP3* HCT116 and LoVo cells compared with that of control (NC) cells by flow cytometry (two-tailed Student’s *t*-test, *P* < 0.0001). **D** Images of xenograft-transplanted nude mouse models (*n* = 3) and dissected tumors 30 days after injection with sh*PTBP3* HCT116 and LoVo cells and their corresponding NC cell lines. **E** Xenograft tumor growth curves of sh*PTBP3* HCT116 and LoVo cells and their corresponding NC cell lines (two-tailed Student’s *t*-test, *P* < 0.05). **F** Xenograft tumor weight of sh*PTBP3* HCT116 and LoVo cells and their corresponding NC cell lines. The results are presented as means ± s.d. and are representative of at least three independent experiments (two-tailed Student’s t-test, *P* < 0.01).

To assess the functional significance of *PTBP3* in CRC cells, we first silenced *PTBP3* expression in HCT116 and LoVo cells with a lentiviral vector carrying *PTBP3*-specific small hairpin RNA (shRNA). Control cells were transfected with a lentiviral vector carrying negative control shRNA. The transfection effect was observed according to green fluorescence protein expression, and the *PTBP3* silencing effect was confirmed by qRT-PCR and WB (Fig. S1a–c). Next, we investigated the role of *PTBP3* in HCT116 and LoVo cells using the Cell Counting Kit-8 (CCK-8) assay and colony formation assay. *PTBP3* silencing reduced cell proliferation activity (Fig. 2A, B). Next, we assessed the effect of *PTBP3* on the cell cycle using flow cytometry. Silencing *PTBP3* expression increased the proportion of cells arrested in the G0/1 phase and decreased the proportion of cells in S phase for both HCT116 and LoVo cells (Fig. 2C). These findings suggested that *PTBP3* plays an essential role in CRC proliferation in vitro. To further evaluate the role of *PTBP3* in vivo, we injected HCT116 and LoVo cells with stable *PTBP3* knockdown and their corresponding NC cells into nude mice. All the mice developed tumors at the injection site (Fig. S2a), but the average size and weight of the tumors generated by *PTBP3* knockdown cells were significantly smaller than those generated by NC cells (Fig. 2D–F). Immunohistochemistry (IHC) showed lower Ki67 expression in the *PTBP3* knockdown tumor tissue group than in the NC group (Fig. S2b), suggesting that *PTBP3* knockdown suppressed the proliferation of cancer cells. All tumor tissues were verified by hematoxylin and eosin (H&E) staining (Fig. S2b). Together, these results confirmed the oncogenic activity of *PTBP3* in CRC in vivo, a finding consistent with that observed in vitro.

### *PTBP3* binds to the *UBE4A* 3′ UTR and may stabilize *UBE4A* mRNA

**Fig. 3:**
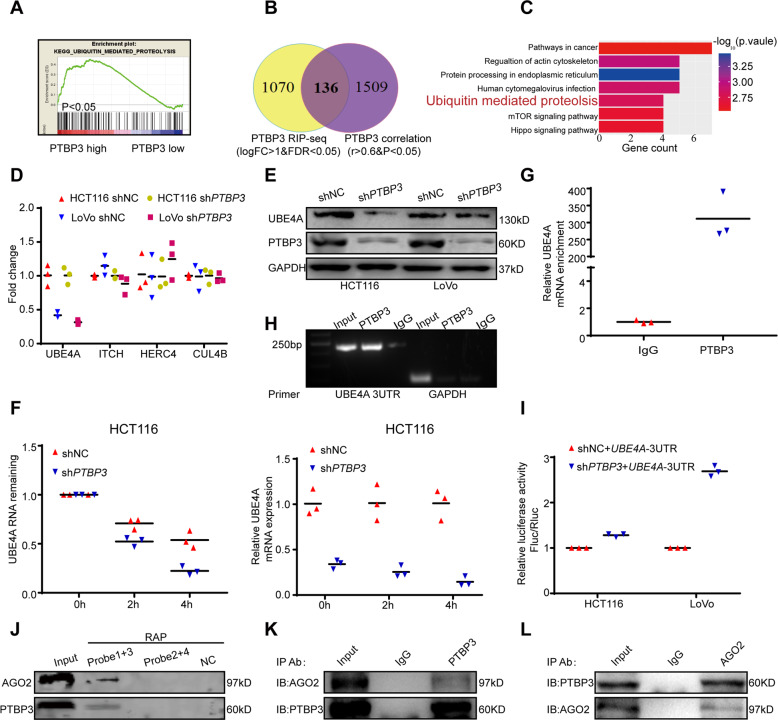
*PTBP3* binds to the *UBE4A* 3′ UTR and stabilizes *UBE4A* mRNA. **A** GSEA plot showing that the *PTBP3* expression level was positively correlated with ubiquitin-mediated proteolysis gene signatures. **B** Venn diagram showing common genes in these two groups (highly correlated genes of *PTBP3* from TCGA and *PTBP3* RIP-seq data). **C** Connected pathways of Kyoto Encyclopedia of Genes and Genomes (KEGG) analysis with common genes. **D** Relative mRNA expression levels of ubiquitin-mediated proteolysis-related genes (*UBE4A*, ITCH, CUL4B, HERC4) in *PTBP3*-silenced HCT116 and LoVo cells compared with those of control (NC) cells (measured by qRT-PCR; GAPDH was used as an internal control) (two-tailed Student’s *t*-test, *P* < 0.05). **E** Relative protein level of UBE4A in *PTBP3*-silenced HCT116 and LoVo cells compared with that of control (NC) cells (measured by Western blotting). **F** Decay rate of mRNA (left) and qPCR (right) analysis of *UBE4A* at the indicated times after actinomycin D (5 µg/ml) treatment in sh*PTBP3* and shNC HCT116 cells (two-tailed Student’s *t*-test, *P* < 0.05). **G** RIP assay showed the real-time PCR of *UBE4A*-3′ UTR enrichment of *PTBP3* compared with IgG in HCT116 cells (two-tailed Student’s *t*-test, *P* < 0.01). **H** RIP assay showed the PCR products of *UBE4A*-3′ UTR and GAPDH enrichment of *PTBP3* compared with IgG in HCT116 cells. (**I**) Relative luciferase activity of the reporter containing the *UBE4A*-3′ UTR cotransfected into *PTBP3*-silenced HCT116 and LoVo cells and their corresponding negative control cells (two-tailed Student’s *t*-test, *P* < 0.01). (**J**)RNA pull-down assay showing that PTBP3 and AGO2 were enriched in *UBE4A*-3′ UTR 1 + 3 probes. **K** Immunoprecipitation assays showing that *PTBP3* bind to AGO2 in HCT116 cells. **L** Immunoprecipitation assays showing that AGO2 bind to PTBP3 in HCT116 cells. The results are presented as means ± s.d. and are representative of at least three independent experiments.

To study the mechanism related to the oncogenic activity action of *PTBP3*, we performed gene set enrichment analysis (GSEA) using mRNA expression data from TCGA CRC datasets (Table S3). *PTBP3* mRNA expression correlated positively with ubiquitin-mediated proteolysis gene signatures, indicating that this pathway is the most closely related to *PTBP3* activity (Fig. 3A). However, in previous studies, *PTBP3* was characterized as an RNA-binding protein without a protein domain that participates in ubiquitylation directly [27]. Therefore, we speculated that *PTBP3* might impact some critical ubiquitylation-related genes to influence the pathway. Consequently, we first analyzed *PTBP3*-correlated genes in TCGA CRC datasets (Table S4) and downloaded *PTBP3* RNA immunoprecipitation sequencing (RIP-seq) data (Table S5) [28]. Next, a Venn diagram was drawn, and 136 closely correlated genes were obtained (Fig. 3B). The 136 genes were then subjected to Kyoto Encyclopedia of Genes and Genomes (KEGG) pathway analysis (Fig. 3C), which identified four genes (*UBE4A*, *ITCH, CUL4B*, and *HERC4*) distributed in the ubiquitin-mediated proteolysis pathway (Fig. S3A). To investigate whether *PTBP3* affected the expression of these four genes, we first performed qRT-PCR in HCT116 and LoVo *PTBP3* knockdown cells. Only the mRNA levels of *UBE4A* were decreased (Fig. 3D). Subsequent WB experiments confirmed this correlation at the protein level (Fig. 3e). A previous study showed that RNA-binding proteins maintain mRNA stability by preventing RISC-mediated mRNA degradation [29, 30]. Similarly, *PTBP3* functions in mRNA decay and mRNA stability [6, 28]. Therefore, we speculated that *PTBP3* might associate with the *UBE4A*-3′ UTR to affect its mRNA stability and expression. To verify this hypothesis, we first assessed the RNA decay rate of *UBE4A* in *PTBP3* knockdown CRC cells and corresponding control cells. *UBE4A* mRNA expression was initially decreased, and the *UBE4A* mRNA half-life was consistently markedly shortened following *PTBP3* knockdown (Fig. 3F). Next, we performed RIP assays in HCT116 cells, and primers targeting the *UBE4A*-3′ UTR were enriched with the anti-*PTBP3* antibody significantly more than that with IgG (Fig. 3G, H). Additionally, we developed a psiCheck-2 reporter plasmid containing the *UBE4A*-3′ UTR sequence cloned downstream of Renilla luciferase (Rluc) and firefly luciferase driven by the HSV-TK promoter. Rluc activity in cells with *PTBP3* knockdown was significantly higher than that in control cells (Fig. 3I). These results suggested that *PTBP3* binds to the *UBE4A*-3′ UTR directly. Next, we designed 4 probes targeting the *UBE4A*-3′ UTR and performed a biotin-labeled RNA Antisense Purification Assay. (Table S1). PTBP3 was present in the products for probes 1 + 3 (Fig. 3j). The presence of AGO2 (a core component of the RISC complex) further proved our speculation (Fig. 3J). These results suggested that *PTBP3* may prevent *UBE4A* degradation by RISC to stabilize its mRNA. To provide more direct evidence of this speculation, we performed immunoprecipitation experiments and revealed interactions between PTBP3 and AGO2 (Fig. 3K, I). Collectively, our results suggested that *PTBP3* stabilizes *UBE4A* mRNA by binding to its 3′ UTR to prevent mRNA degradation mediated by the AGO2-containing RISC complex.

### *UBE4A* promotes CRC cell proliferation in vitro

**Fig. 4:**
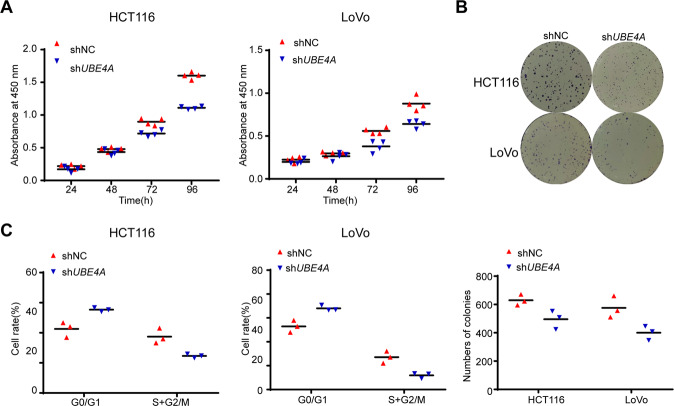
*UBE4A* promotes CRC cell proliferation in vitro. **A** Reduction in the proliferation ability of sh*UBE4A* HCT116 and LoVo cells compared with that of control (NC) cells using the CCK8 assay (two-way ANOVA, *P* < 0.01). **B** Reduction in the colony formation ability of sh*UBE4A* HCT116 and LoVo cells compared with that of control (NC) cells using the colony formation assay. The bar graph indicates the number of colonies (two-tailed Student’s *t*-test, *P* < 0.05). **C** Cell cycle distribution of sh*UBE4A* HCT116 and LoVo cells compared with that of control (NC) cells was analyzed by flow cytometry. The results are presented as means ± s.d. and are representative of at least three independent experiments (two-tailed Student’s *t*-test, *P* < 0.05).

The above results indicated that *PTBP3* regulates oncogenic activity in CRC and regulates *UBE4A* expression by binding to its 3ʹ UTR. However, the function of *UBE4A* in CRC remains unknown, and no previously published article is available concerning *UBE4A* in CRC. To assess the function of *UBE4A* in CRC cells, we silenced *UBE4A* expression in HCT116 and LoVo cells with a lentiviral vector carrying *UBE4A*-specific shRNA. Control cells were transfected with a lentiviral vector carrying negative control shRNA. The transfection effect was observed by detecting green fluorescence (Fig. S4a), and the *UBE4A* silencing effect was confirmed by qRT-PCR and WB (Fig. S4b, c). Next, we used CCK-8 and colony formation assays to determine the role of *UBE4A* in mediating the malignant behavior of HCT116 and LoVo cells. The results showed that *UBE4A* silencing reduced cell proliferation activity (Fig. 4A, B), a finding that was consistent with the effect of *PTBP3* in CRC. Similarly, we assessed the effect of *UBE4A* on the cell cycle using flow cytometry. Silencing *UBE4A* expression increased the proportion of cells arrested in the G0/1 phase and decreased the proportion of cells in S phase for both HCT116 and LoVo cells (Fig. 4C). These findings suggested that *UBE4A* promotes CRC cell proliferation in vitro, a finding that is consistent with the role of *PTBP3* in CRC.

### *PTBP3* regulates *P53* expression by facilitating ubiquitin-mediated *UBE4A* degradation

**Fig. 5:**
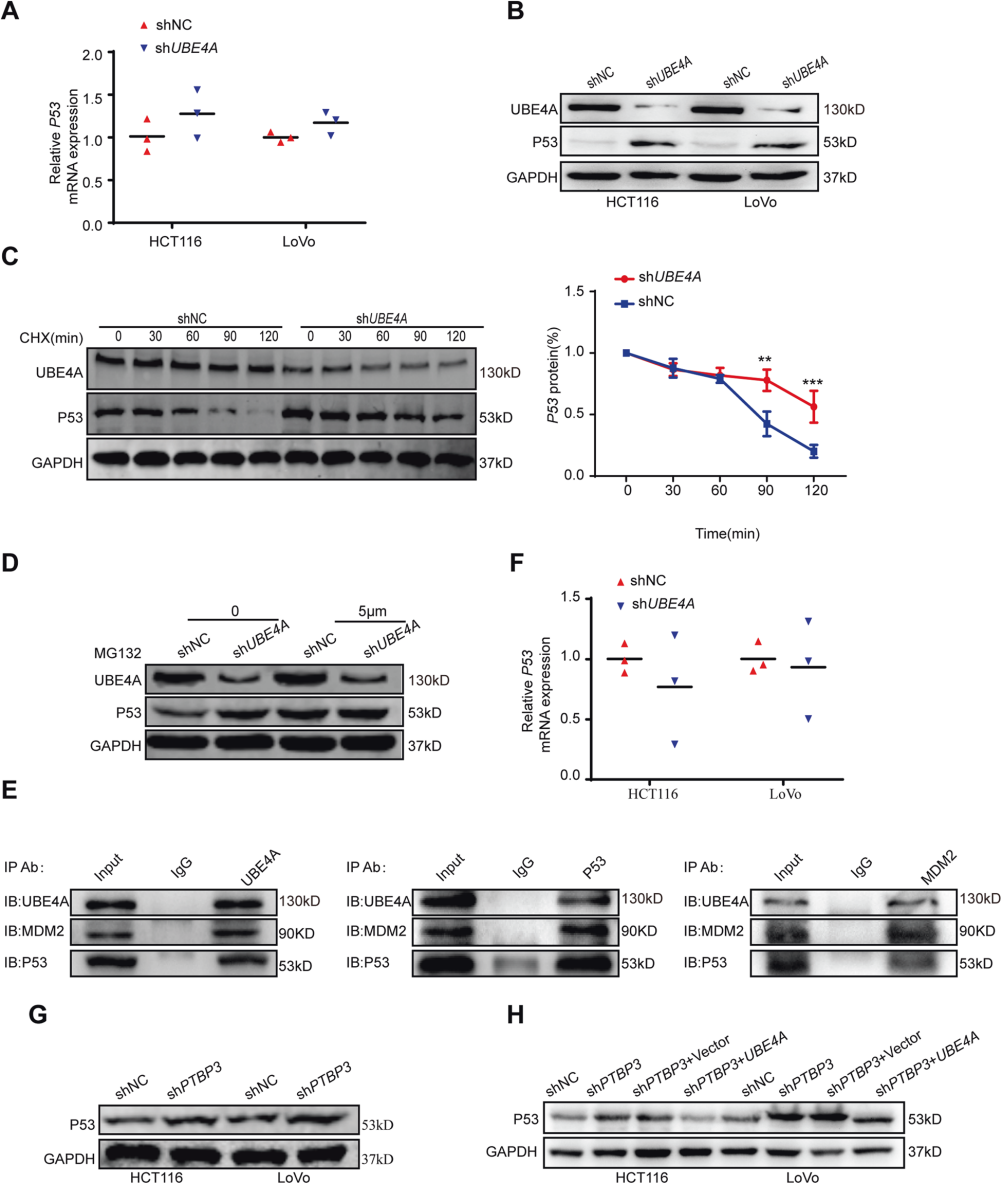
*PTBP3* regulates *P53* expression by *UBE4A* ubiquitin degradation. **A** Relative mRNA expression level of *P53* in *UBE4A*-silenced HCT116 and LoVo cells compared with that of control (NC) cells (measured by qRT-PCR; GAPDH was used as an internal control) (two-tailed Student’s *t*-test, *P* > 0.05). **B** Relative protein level of P53 in *UBE4A*-silenced HCT116 and LoVo cells compared with that of control (NC) cells (measured by Western blotting). **C** sh*UBE4A* and shNC HCT116 cells were treated with 100 μg/mL of cycloheximide (CHX) and harvested at the indicated time points. P53 protein was detected by western blotting (left), quantified by densitometry, and plotted against time to determine P53 stability (right) (two-tailed Student’s *t*-test, *P* < 0.05). **D** sh*UBE4A* and shNC HCT116 cells were treated with 5 μmol/L of MG132 for 12 h, and P53 protein was detected by western blotting. **E** Immunoprecipitation assays showing that UBE4A、MDM2 and P53 three proteins bound to each other. **F** Relative mRNA expression level of P53 in *PTBP3*-silenced HCT116 and LoVo cells compared with that of control (NC) cells (measured by qRT-PCR; GAPDH was used as an internal control) (two-tailed Student’s *t*-test, *P* > 0.05). **G** Relative protein level of P53 in *PTBP3*-silenced HCT116 and LoVo cells (measured by Western blotting). **H** *PTBP3* knockdown HCT116 and LoVo cells were transfected with the *UBE4A* plasmid, and P53 expression was detected. (measured by Western blotting). The results are presented as means ± s.d. and are representative of at least three independent experiments.

Previous studies have shown that the U-box catalytic domain of *UBE4B* is closely related to the RING-finger domain of *MDM2* and is responsible for its E3 activity, and these two enzymes are considered significant regulators of *P53* through the ubiquitination process [17, 31–33]. *UBE4A*, as a homolog of *UBE4B* [17], may also exert the same effect on *P53*. To verify this claim, we performed qRT-PCR and WB analysis in *UBE4A* knockdown CRC cell lines and their corresponding negative control cell lines. *UBE4A* knockdown had no effect on *P53* expression at the mRNA level but significantly increased P53 expression at the protein level (Fig. 5A, B), suggesting that *UBE4A* affects P53 protein stability. To verify this finding, we treated the HCT116 *UBE4A* knockdown cell line and corresponding negative control cell line with cycloheximide (CHX), a protein synthesis inhibitor. P53 expression was increased after *UBE4A* knockdown (Fig. 5C, left), and the rate of P53 degradation was slow at the indicated time after *UBE4A* knockdown (Fig. 5C, right). The stability of P53 was increased by *UBE4A* knockdown (Fig. 5C). Next, we treated the HCT116 *UBE4A* knockdown cell line and corresponding negative control cell line with MG132, a proteasome inhibitor. P53 expression was increased after MG132 treatment, suggesting that *UBE4A* affected P53 stability in the context of proteasome-dependent degradation (Fig. 5d). Immunoprecipitation experiments using the anti-*UBE4A* antibody, anti-*P53* antibody and anti-*MDM2* antibody revealed that the three proteins bound to each other (Fig. 5E). Collectively, these results proved our speculation that *UBE4A* may affect P53 stability via *MDM2-P53* proteasome-dependent degradation to influence CRC proliferation. *PTBP3* regulates P53 expression in hepatocellular carcinoma, but the specific mechanism remains unclear [7]. Here, we performed qRT-PCR and WB analysis of *PTBP3* knockdown CRC cell lines, revealing that *PTBP3* knockdown had no effect on the *P53* mRNA levels but increased P53 protein expression (Fig. 5F, G). Therefore, we speculated that *PTBP3* modulates P53 expression by mediating *UBE4A* expression. Next, we transfected *UBE4A* plasmids into *PTBP3* knockdown HCT116 and LoVo cells (Fig. S4d, e). WB assays showed that *UBE4A* overexpression restored P53 expression in *PTBP3* knockdown HCT116 and LoVo cells (Fig. 5H). Given that LoVo and HCT116 cells possess the wild-type (WT) *P53* gene, we next used the SW480 cell line with mutant *P53* to examine the effect of *PTBP3* and *UBE4A* on CRC. We first silenced *PTBP3* and *UBE4A* in SW480 cells separately. Transfection was detected using green fluorescence (Fig. S5a), and silencing was confirmed by qRT-PCR and WB (Figs. S5b, 5E). Next, we assessed cell viability and P53 expression in SW480 cells. Knockdown of either *PTBP3* or *UBE4A* did not decrease SW480 cell viability or P53 expression (Fig. S5c–e). Collectively, these findings suggest that *PTBP3* regulates WT P53 expression by facilitating the ubiquitin-mediated degradation of *UBE4A* in CRC.

### *UBE4A* overexpression restores *PTBP3* knockdown-mediated CRC cell proliferation

**Fig. 6:**
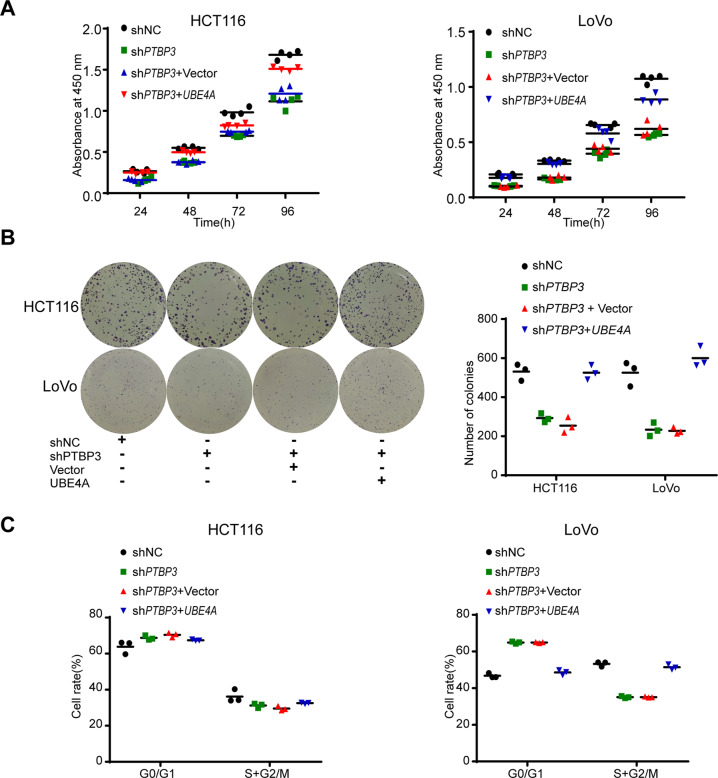
Overexpression of *UBE4A* restores cell proliferation in *PTBP3* knockdown CRC cells. **A** *PTBP3* knockdown HCT116 and LoVo cells were transfected with the *UBE4A* plasmid, and the proliferation ability was quantified using the CCK-8 assay (two-way ANOVA, *P* < 0.001). **B** *PTBP3* knockdown HCT116 and LoVo cells were transfected with the *UBE4A* plasmid, and the colony formation ability was quantified using the colony formation assay (two-tailed Student’s *t*-test, *P* < 0.01). **C** *PTBP3* knockdown HCT116 and LoVo cells transfected with the *UBE4A* plasmid were analyzed by flow cytometry. The results are presented as means ± s.d. and are representative of at least three independent experiments (two-tailed Student’s *t*-test, *P* < 0.05).

To investigate the function of *UBE4A* in the *PTBP3*-mediated promotion of CRC proliferation, we transfected *UBE4A* plasmids into *PTBP3* knockdown HCT116 and LoVo cells and examined the effect of *UBE4A* overexpression on proliferation activity and cell cycle distribution. *UBE4A* overexpression restored the proliferative activity and abnormal cell cycle of *PTBP3* knockdown HCT116 and LoVo cells (Fig. 6A–C). These results further proved that *PTBP3* promotes CRC cell proliferation by regulating *UBE4A*.

## Discussion

*PTBP3* is a protein-coding gene that plays an essential role in humans [34]. Recent studies have shown that *PTPB3* is dysregulated in multiple tumors and plays vital roles in carcinogenesis [6–10], but few studies have elucidated the specific mechanisms, particularly in CRC. In the present study, we found that *PTBP3* is upregulated in CRC patients with a poor prognosis and promotes CRC cell proliferation by meditating *UBE4A* mRNA stability to regulate P53 expression.

In the present study, we first analyzed the TCGA and GEO online databases. *PTBP3* mRNA was upregulated in CRC. Next, we performed an assay using clinical tissues to further confirm this conclusion at both the mRNA and protein levels. Next, we analyzed the correlations between the *PTBP3* expression level and clinical features and found that a high *PTBP3* expression level was correlated with clinical features, particularly the tumor size. Ping et al.’s study also showed that *PTBP3* is upregulated and associated with a poorer clinical prognosis [9], supporting our findings. Additionally, *PTBP3* overexpression was found in hepatocellular cancer [7], gastric cancer [8], and breast cancer [6] and was correlated with a poor clinical prognosis, suggesting that *PTBP3* may serve as an oncogene in multiple cancers. However, these studies, including ours, lacked a sufficient amount of data to draw reliable conclusions. Thus, further multicenter clinical trials are required. To further investigate the oncogenic role of *PTBP3* in CRC, we silenced *PTBP3* in HCT116 and LoVo cells. After *PTBP3* knockdown, CRC cell proliferation was significantly inhibited in HCT116 and LoVo cell lines in vivo and in vitro. This finding was partly consistent with that reported by Ping et al [9], who used HCT116 and SW480 cells for research but only conducted the CRC cell proliferation assay in HCT116 cells and not in SW480 cells. Considering that *PTBP3* regulated P53 expression in our study, we also silenced *PTBP3* in the SW480 cell line, a *TP53*-mutant gene cell line. However, silencing *PTBP3* in SW480 cells had no effect on CRC cell proliferation. Similarly, *PTBP3* has no effect on gastric cancer proliferation but promotes breast cancer and hepatocellular cancer growth [6, 7]. We speculated that *PTBP3*-mediated regulation of P53 expression might be an explanation. Mechanistically, we found that *PTBP3* was positively correlated with the ubiquitin-mediated proteolysis signaling pathway according to GSEA. However, *PTBP3* does not have a protein domain that participates in ubiquitination [27]. Therefore, we investigated four candidate genes related to the ubiquitin-mediated proteolysis signaling pathway by *PTBP3* RIP-seq and identified *PTBP3*-correlated genes using TCGA database analysis. *PTBP3* knockdown decreased *UBE4A* expression at both the mRNA and protein levels. We hypothesized that *PTBP3* may influence *UBE4A* mRNA stability. We examined the decay rate of *UBE4A* mRNA, the results of which proved our hypothesis. Next, we performed RIP and luciferase reporter assays to confirm *PTBP3* binding to the *UBE4A* 3′ UTR, and the RAP assay further proved this finding. The presence of *AGO2*, a core component of RISC, in the RAP assay suggested that *PTBP3* may stabilize *UBE4A* mRNA by preventing RISC-mediated degradation of *UBE4A* mRNA. Further immunoprecipitation assays showed that PTBP3 and AGO2 could bind to each other. Collectively, our results first proved that *PTBP3* has a marked impact on the ubiquitin-mediated proteolysis signaling pathway by stabilizing *UBE4A* mRNA in CRC.

The ubiquitin-mediated proteolysis signaling pathway plays a role in every cellular function, including essential processes for carcinogenesis, such as proliferation, apoptosis, and angiogenesis, which commonly occur in CRC [35–37]. *UBE4A*, an E3 ubiquitin ligase, has seldom been reported in the context of tumorigenesis. *UBE4A* represses ILEI protein expression to inhibit prostate cancer progression [38]. However, in thyroid carcinoma, *UBE4A* was reported as a ubiquitin ligase that is inversely correlated with PCBP1 protein expression and promotes cancer progression [19]. Therefore, the specific function and mechanism of CRC must be addressed. In the present study, we first demonstrated that *UBE4A* knockdown promoted CRC proliferation and increased P53 protein expression in CRC cell lines expressing the WT *P53* gene but not in CRC cell lines expressing the mutant *P53* gene. Further experiments indicated that *UBE4A* might influence P53 stability via the *MDM2-P53* pathway. However, we did not elucidate the specific mechanism concerning the relationship between *UBE4A* and mutant P53 expression. Additionally, *PTBP3* knockdown increased WT P53 expression, and a similar finding was reported in hepatocellular carcinoma [7]. However, *PTBP3* knockdown decreased *UBE4A* expression in CRC cell lines with both WT and mutant *P53*, suggesting that *PTBP3*-regulated *UBE4A* stability is a common phenomenon in CRC. Finally, we overexpressed *UBE4A* in *PTBP3* knockdown CRC cells, revealing that *UBE4A* restored P53 expression and inhibited CRC cell proliferation. Collectively, these results suggested that *PTBP3* mediates *UBE4A* mRNA stability to regulate WT P53 expression and promote CRC proliferation.

## Conclusions

In summary, our results revealed that *PTBP3* is overexpressed and correlated with a poor prognosis and plays an oncogenic role, contributing to CRC proliferation. Additionally, *PTBP3* is closely related to the ubiquitin-mediated proteolysis signaling pathway-related gene *UBE4A* and may mediate its mRNA stability to regulate its expression. However, we demonstrate for the first time that *UBE4A* knockdown inhibits CRC proliferation, which may be associated with P53 stability. In conclusion, our results indicate that the *PTBP3*/*UBE4A*/P53 axis may be a prognostic marker and therapeutic target in CRC, potentially providing new insight into CRC progression and treatment.

## Supplementary information

aj-checklist

Supplementary information

Figure S1

Figure S2

Figure S3

Figure S4

Figure S5

Table S1

Table S2

Table S3

Table S4

Table S5

## Data Availability

All the data generated or analyzed during this study are included in this published article [and its supplementary information files].
