# Supplementary material for: PTBP3 modulates P53 expression and promotes colorectal cancer cell proliferation by maintaining UBE4A mRNA stability
Source: Cell Death Dis. 2022 Feb 8;13(2):128. doi: 10.1038/s41419-022-04564-8 (PMC8826374; doi:10.1038/s41419-022-04564-8)
Supplement: Supplementary file 2 — Supplementary information [file 41419_2022_4564_MOESM2_ESM.doc]

**Supplementary Information**

**Additional file 1:Fig. S1.** Stably knocked down PTBP3 in HCT116 and LoVo cell lines. **(A)** Representative images of HCT116 and LoVo cell lines after transfection with a UBE4A-specific small hairpin RNA (shUBE4A) and its corresponding lentiviral vector carrying negative control shRNA (shNC). **(B)** Relative mRNA expression level of UBE4A in HCT116 and LoVo cells transfected with a lentiviral vector carrying PTBP3-specific small hairpin RNA (shPTBP3) and its corresponding lentiviral vector carrying negative control shRNA (shNC) (measured by qRT-PCR; GAPDH was used as an internal control) (two-tailed Student’s t-test, p < 0.01). **(C)** Relative protein level of PTBP3 in HCT116 and LoVo cells transfected with a lentiviral vector carrying a PTBP3-specific small hairpin RNA (shPTBP3) and its corresponding lentiviral vector carrying negative control shRNA (shNC) (measured by western blotting). The results are presented as mean±s.d. and are representative of at least three independent experiments.

**Additional file 2:Fig. S2.** Xenograft tumors of PTBP3 knockdown in HCT116 and LoVo cell lines. **(A)** Image of the injected mouse xenograft tumors. **(B)** Representative images of H&E staining of mouse xenograft tumors and PTBP3 and Ki67 IHC of mouse xenograft tumors (scale bar, 20 µm).

**Additional file 3:Fig. S3.** PTBP3 correlated genes in the ubiquitin mediated proteolysis pathway. (A) Correlation of PTBP3 with four ubiquitin-mediated proteolysis genes.

**Additional file 4:Fig. S4.** Stable knockdown of UBE4A and transiently transfected plasmid UBE4A in HCT116 and LoVo cell lines. **(A)** Representative images of HCT116 and LoVo cell lines after transfection with a UBE4A-specific small hairpin RNA (shUBE4A) and its corresponding lentiviral vector carrying negative control shRNA (shNC). **(B)** Relative mRNA expression level of UBE4A in HCT116 and LoVo cells transfected with a lentiviral vector carrying UBE4A-specific small hairpin RNA (shUBE4A) and its corresponding lentiviral vector carrying negative control shRNA (shNC) (measured by qRT-PCR; GAPDH was used as an internal control) (two-tailed Student’s t-test, p < 0.01). **(C)** Relative protein level of UBE4A in HCT116 and LoVo cells transfected with a lentiviral vector carrying UBE4A-specific small hairpin RNA (shUBE4A) and its corresponding lentiviral vector carrying negative control shRNA (shNC) (measured by Western blotting). **(D)** Relative mRNA expression level of UBE4A in HCT116 and LoVo cells transfected with UBE4A and vector plasmids (measured by qRT-PCR; GAPDH was used as an internal control) (two-tailed Student’s t-test, p < 0.0001). **(E)** Relative protein level of UBE4A in HCT116 and LoVo cells transfected with UBE4A and vector plasmids (measured by Western blotting). The results are presented as means±s.d. and are representative of at least three independent experiments.

**Additional file 5:Fig. S5.** Knockdown of PTBP3 and UBE4A separately in the SW480 cell line had no effect on cell proliferation or p53 expression. **(A)** Representative images of SW480 cell line after transfection with PTBP3-specific small hairpin RNA (shPTBP3)、UBE4A-specifific small hairpin RNA (shUBE4A) and their corresponding lentiviral vectors carrying negative control shRNA (shNC).**（B）**Relative mRNA expression level of PTBP3 and UBE4A in the SW480 cell line after transfection with PTBP3-specific small hairpin RNA (shPTBP3)、UBE4A-specifific small hairpin RNA (shUBE4A) and their corresponding lentiviral vectors carrying negative control shRNA (shNC) (two-tailed Student’s t-test, p < 0.01). **(C)** Colony formation ability of shPTBP3 SW480 and shUBE4A SW480 cells compared with that of control (NC) cells using the colony formation assay. The bar graph indicates the number of colonies (two-tailed Student’s t-test, p >0.05). **(D)** Proliferation ability of shPTBP3 SW480 and shUBE4A SW480 cells compared with that of control (NC) cells using the CCK-8 assay (two-way ANOVA, p >0.05). **(E)** Relative protein levels of UBE4A, PTBP3, and P53 in shPTBP3 SW480 and shUBE4A SW480 cells compared with those of control (NC) cells. The results are presented as means±s.d. and are representative of at least three independent experiments.

**Additional file 6: Table S1:** Primers and probe sequences used in the study

**Additional file 7: Table** **S2:** Antibodies used in the study

**Additional file 8: Table S3:** GSEA of PTBP3 using TCGA colorectal cancer datasets

**Additional file 9: Table S4:** Correlation gene analysis of PTBP3 using TCGA colorectal cancer datasets

**Additional file 10: Table S5:** PTBP3 rip-seq data downloaded from an article[26]
