# Supplementary figures and images for: PTBP3 modulates P53 expression and promotes colorectal cancer cell proliferation by maintaining UBE4A mRNA stability
Source: Cell Death Dis. 2022 Feb 8;13(2):128. doi: 10.1038/s41419-022-04564-8 (PMC8826374; doi:10.1038/s41419-022-04564-8)

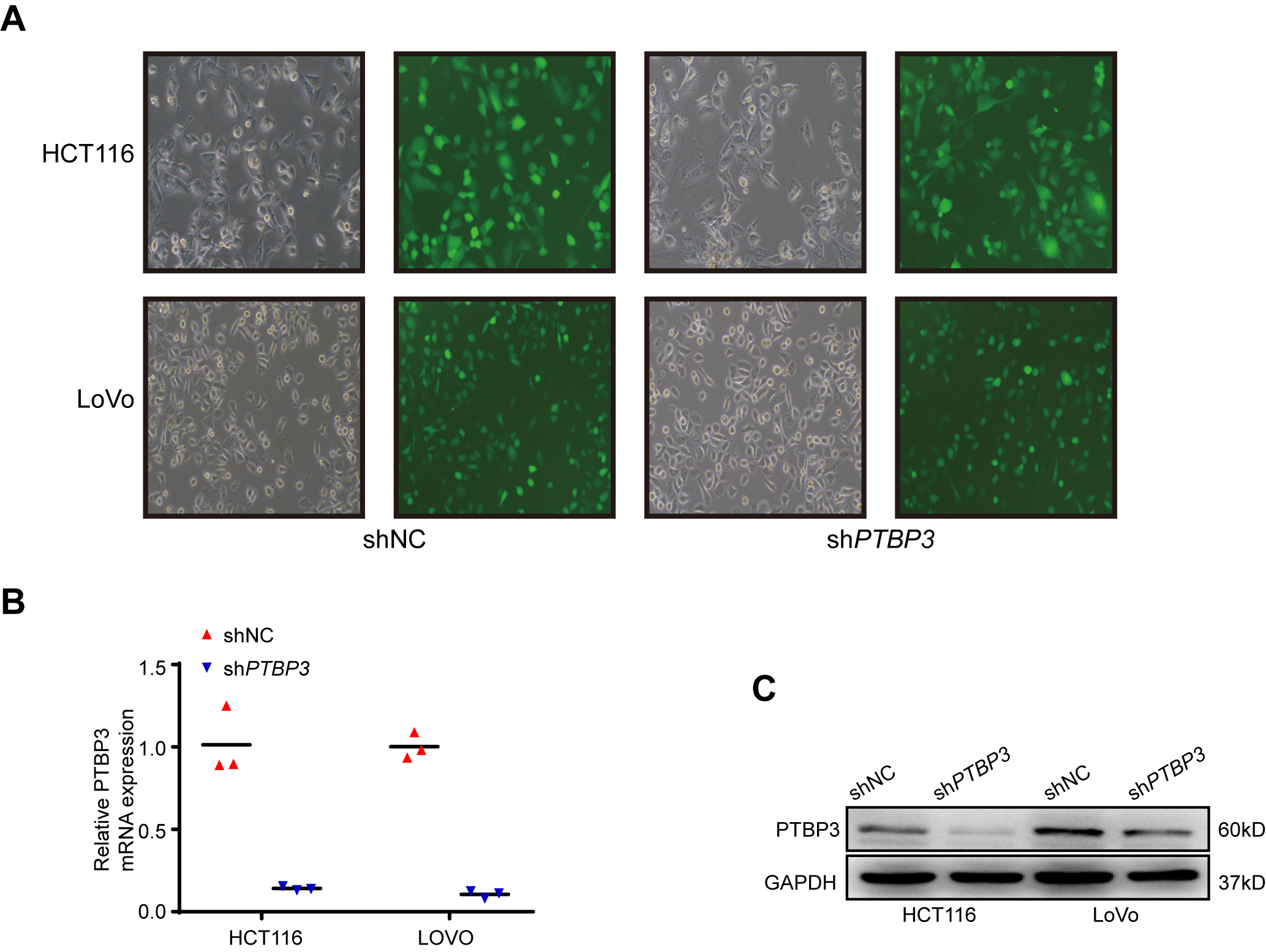

Supplement: Supplementary file 3 — Figure S1 [file 41419_2022_4564_MOESM3_ESM.tif]

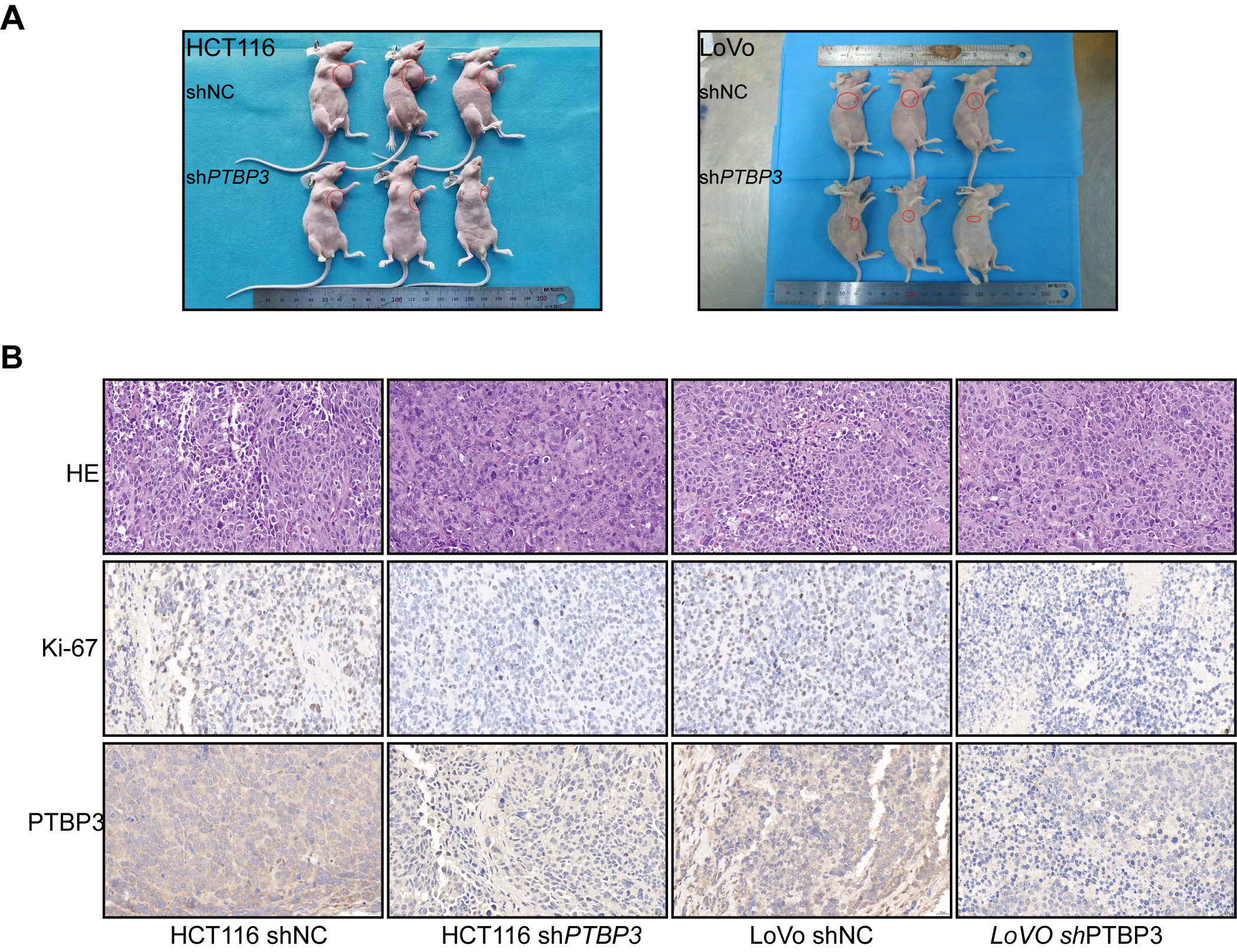

Supplement: Supplementary file 4 — Figure S2 [file 41419_2022_4564_MOESM4_ESM.tif]

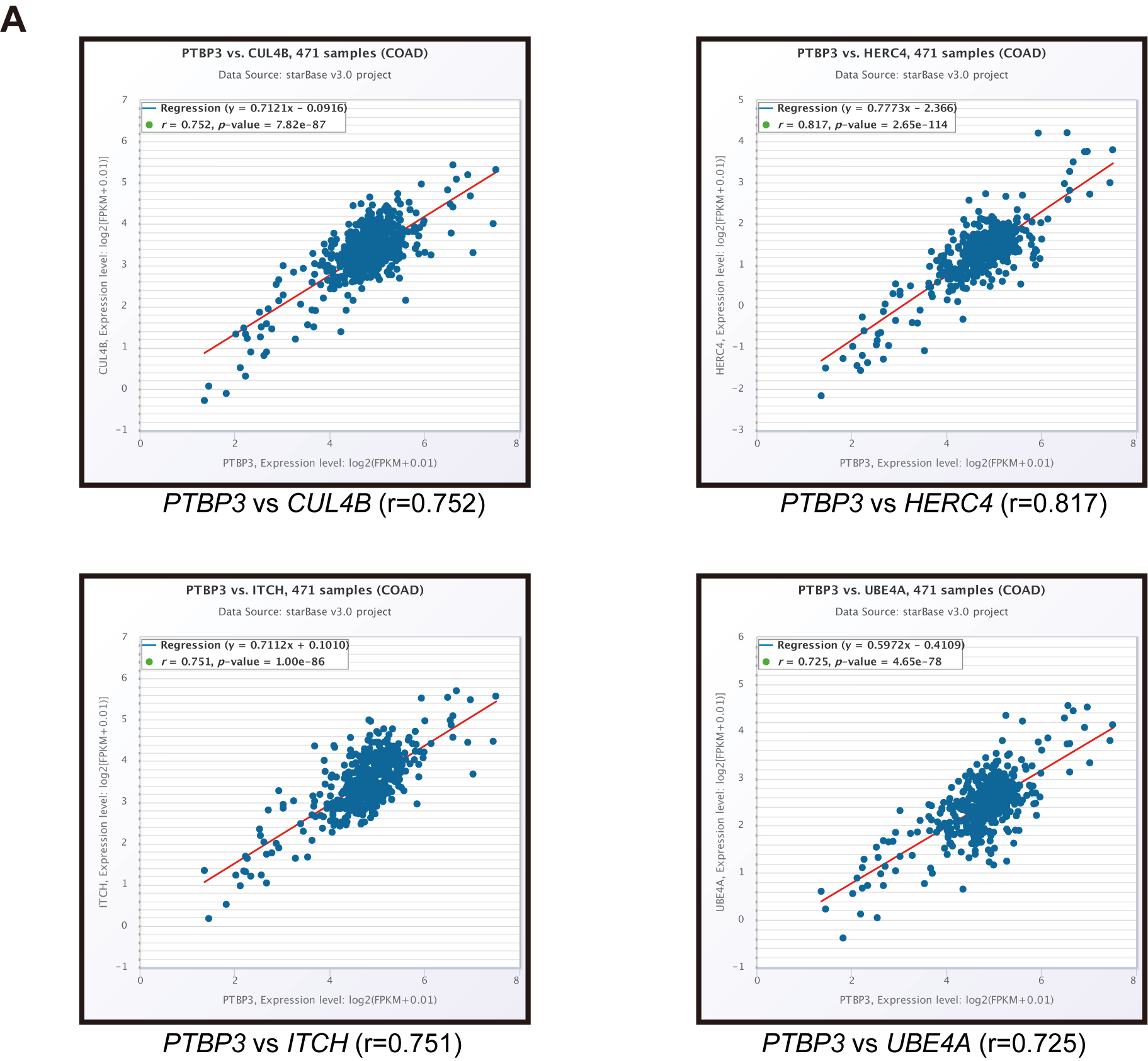

Supplement: Supplementary file 5 — Figure S3 [file 41419_2022_4564_MOESM5_ESM.tif]

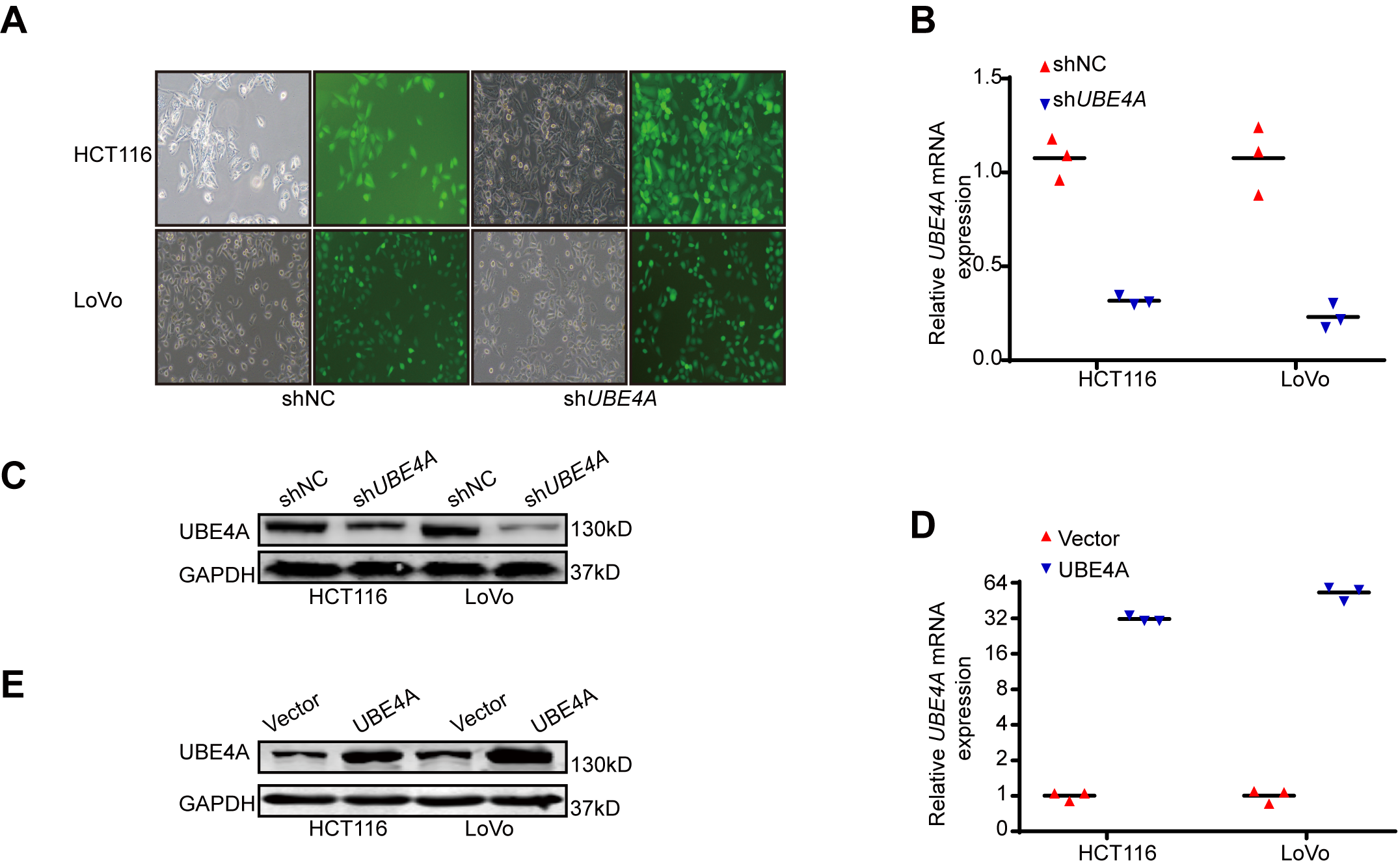

Supplement: Supplementary file 6 — Figure S4 [file 41419_2022_4564_MOESM6_ESM.tif]

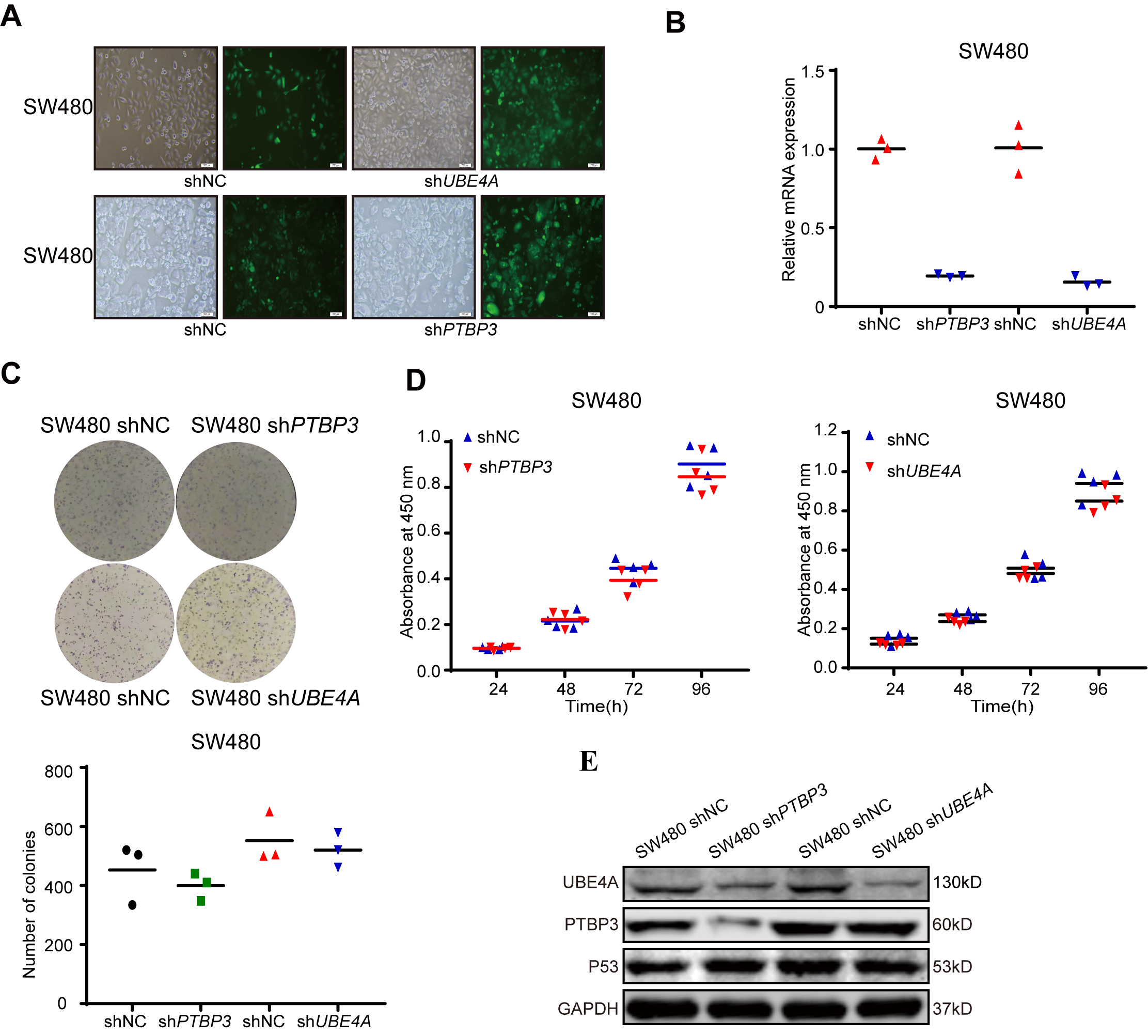

Supplement: Supplementary file 7 — Figure S5 [file 41419_2022_4564_MOESM7_ESM.tif]
