# Supplementary material for: PTBP3 modulates P53 expression and promotes colorectal cancer cell proliferation by maintaining UBE4A mRNA stability
Source: Cell Death Dis. 2022 Feb 8;13(2):128. doi: 10.1038/s41419-022-04564-8 (PMC8826374; doi:10.1038/s41419-022-04564-8)
Supplement: Supplementary file 8 — Table S1 [file 41419_2022_4564_MOESM8_ESM.doc]

**Supplementary Table 1. primers and shRNA sequence**

| **Sequence** | **Target** | **Sense/Forward** | **Antisense/Reverse** |
| --- | --- | --- | --- |
| shRNA | human PTBP3 | CCAAGGTCTGACTAAGGATTT | N/A |
| shRNA | human LV3-NC | TTCTCCGAACGTGTCACGT | N/A |
| shRNA | human UBE4A | GCTGGGAGTAATTCTGAGTAT | N/A |
| shRNA | human LV3-NC | TTCTCCGAACGTGTCACGT | N/A |
| qPCR primers | human PTBP3 | CAAGCTTGACACCAGGGGTCTGGACTTA | CGGGATCCTATGGTCCAGTTTTAGGAGA |
| qPCR primers | Human UBE4A | TGTAAAACGACGGCCAGT | CAGGAAACAGCTATGACC |
| qPCR primers | human HERC4 | TGTAAAACGACGGCCAGT | CAGGAAACAGCTATGACC |
| qPCR primers | human ITCH | TGTAAAACGACGGCCAGT | CAGGAAACAGCTATGACC |
| qPCR primers | human CUL4B | TGTAAAACGACGGCCAGT | CAGGAAACAGCTATGACC |
| qPCR primers | human TP53 | CAGCACATGACGGAGGTTGT | TCATTCCAAATACTCCACGC |
| qPCR primers | human GAPDH | TGCACCACCAACTGCTTAGC | GGCATGGACTGTGGTCATGAG |
| qPCR primers | human UBE4A 3UTR | GGGACATTCCCCTATGGATT | TTTGAGGCTTTCCAGCTGAT |
| UBE4A probe1 | human UBE4A 3UTR | TTGTTAACAAACACCAAAGA |  |
| UBE4A probe2 | human UBE4A 3UTR | TAGTGATTAAAAGTGGGAGA |  |
| UBE4A probe3 | human UBE4A 3UTR | GACAATAGGACACATAAATG |  |
| UBE4A probe4 | human UBE4A 3UTR | TCAGAAAAATTACTACTAAC |  |
