# Supplementary material for: PTBP3 modulates P53 expression and promotes colorectal cancer cell proliferation by maintaining UBE4A mRNA stability
Source: Cell Death Dis. 2022 Feb 8;13(2):128. doi: 10.1038/s41419-022-04564-8 (PMC8826374; doi:10.1038/s41419-022-04564-8)
Supplement: Supplementary file 9 — Table S2 [file 41419_2022_4564_MOESM9_ESM.docx]

**Supplementary Table 2. Primary and secondary antibodies**

| Antibody | Host | Vendor |
| --- | --- | --- |
| anti-PTBP3 | Mouse | Santa Cruz, #A1519 |
| anti-UBE4A | Rabbit | Sino Biological, #101019-T36 |
| anti-TP53 | Rabbit | Proteintech, #10442-1-AP |
| anti-AGO2 | Rabbit | Proteintech, #10686-1-AP |
| anti-GAPDH | Mouse | Proteintech, #60004-1-Ig |
| anti-Ki67 | Rabbit | Servicebio,#GB111499 |
| anti-MDM2 | Rabbit | Affinit Biosciences,#AF0208 |
| IRDye 800CW anti-Rabbit IgG | Goat | LI-COR Biosciences, 925-32210 |
| IRDye 800CW anti-Mouse IgG | Goat | LI-COR Biosciences, 925-32211 |
| HRP anti-Rabbit IgG | Goat | Servicebio,#GB23303 |
